# The Clinical Impact of Early Steroid Withdrawal on Diabetes Mellitus After Liver Transplantation: A Population-Based Cohort Study

**DOI:** 10.3389/ti.2026.15432

**Published:** 2026-02-12

**Authors:** Haeseon Lee, YoungRok Choi, Hae Sun Suh

**Affiliations:** 1 Department of Pharmacotherapy, College of Pharmacy, University of Utah, Salt Lake City, UT, United States; 2 Institute of Regulatory Innovation Through Science (IRIS), Kyung Hee University, Seoul, Republic of Korea; 3 Seoul National University Hospital, College of Medicine, Seoul National University, Seoul, Republic of Korea; 4 College of Pharmacy, Kyung Hee University, Seoul, Republic of Korea

**Keywords:** corticosteroid withdrawal, immunosuppression, landmark analysis, liver transplantation, post-transplant diabetes mellitus

## Abstract

Despite the metabolic risks associated with corticosteroids after liver transplantation (LT), the optimal timing for their withdrawal remains uncertain due to limited and inconsistent evidence. To evaluate the impact of corticosteroid withdrawal timing on the development of *de-novo* post-transplant diabetes mellitus (PTDM), we performed a retrospective cohort study of 6,295 adult recipients who underwent LT between 2009 and 2021 in South Korea, utilizing a national health insurance claims database. A landmark analysis with time-varying propensity score matching was conducted at one-, three-, and six-month post-transplantation to compare the incidence of PTDM between steroid withdrawal and maintenance groups. Early steroid withdrawal within 3 months significantly reduced PTDM risk (HR = 0.586; 95% CI = 0.407–0.846 at 1 month, HR = 0.766; 95% CI = 0.611–0.960 at 3 month), whereas withdrawal after 3 months showed no significant benefit (HR = 0.844; 95% CI = 0.619–1.152 at 6 month). Rejection events were rare, suggesting no substantial compromise in graft function. These findings indicate that corticosteroid withdrawal within the first three months post-LT can lower the risk of PTDM without increasing rejection risk, supporting timely steroid tapering as part of post-transplant immunosuppressive strategies to reduce long-term metabolic complications.

## Graphical Abstract

**Figure:**
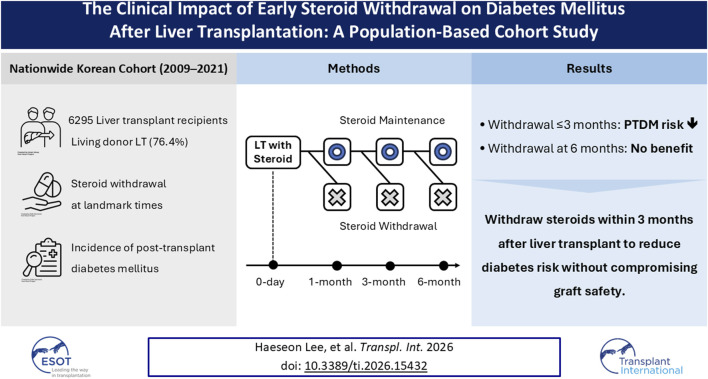

## Introduction

Liver transplantation (LT) has become the most effective treatment for patients with liver cancer or end-stage liver diseases [1], with 5-year graft survival rate exceeding 75% [2]. Long-term care after LT encompasses the management of hypertension, diabetes, bone health, and cancer surveillance [1, 3], underscoring the importance of reducing preventable complications such as post-transplant diabetes mellitus (PTDM) [4]. PTDM affects 30%–40% of recipients and increases the risks of infection, mortality, and cardiovascular disease, the leading cause of non-graft-related death after transplantation [5–7].

Steroids are the primary modifiable risk factor for PTDM due to their multiple interactions within glucose metabolism [8, 9]. While traditionally essential for induction of immunosuppression and rejection control [10], their prolonged use has come under critical review. The 2018 International Liver Transplantation Society Consensus Statement recommended minimizing steroids use to prevent metabolic complications [11]. Many centers empirically adopt steroid-tapering protocols [12], often around 3 months [13], but robust evidence and formal guidelines on optimal timing remain limited.

Given the complex nature of diabetes and the heterogeneity of transplant care, population-based studies reflecting real-world practice are crucial [14]. South Korea, with one of the highest global rates of living donor LT [15], provides a valuable setting to investigate LT outcomes across diverse clinical scenarios. Using nationwide healthcare insurance data, we aimed to evaluate the association between the timing of steroid withdrawal after LT and PTDM without compromising the liver graft function in liver recipients.

## Methods

### Study Design and Population

This retrospective cohort study utilized the Health Insurance Review and Assessment Service database from South Korea, which includes comprehensive information on patient characteristics, diagnoses, treatments, prescription, and medical expenses for the entire population of approximately 50 million people [16]. Patients aged >18 years who received LT between 2009 and 2021, with initial steroid-containing immunosuppressive regimen, were included and followed up until December 2021. Liver recipients were identified as those with an electronic data interchange (EDI) code related to LT for health insurance reimbursement (EDI code of Q80 for cadaver donor LT and Q81 for living donor LT). Patients were excluded if they had a history of diabetes mellitus within 1 year prior to LT. Furthermore, patients requiring continuous steroid use for specific conditions (e.g., autoimmune liver disease) were excluded, irrespective of the timing of diagnosis. This exclusion was implemented to delineate steroid exposure attributable to the immunosuppressive regimen from steroid use for the management of other medical conditions [17, 18]. All diagnoses were identified using ICD-10-CM codes (Supplementary Table S1). This study was approved by the Institutional Review Board of Kyung Hee University (No. KHSIRB-22-176(EA)) and was presented according to Strengthening the Reporting of Observational Studies in Epidemiology reporting guideline [19].

### Landmark Analyses Based on Steroid Use

To assess the potential impact of steroid withdrawal at various time points after LT, we employed a landmark approach to facilitate the identification of causal effects, adhering to the foundational assumptions of propensity score analyses [20]. We set 1-, 3-, and 6-month post-transplantation as landmark times. At each time point, the patients were categorized into ‘steroid withdrawal’ or ‘steroid maintenance’ groups according to their steroid use status from the previous landmark time to the current landmark time. We calculated propensity score using logistic regression models at each landmark time point based on the latest covariates, including comorbidities [21] and hepatitis C virus infections [22], alongside demographics and immunosuppressive regimen. Subsequently, we performed 1:1 matching of patients from the steroid withdrawal group with those from the steroid maintenance group, creating two cohorts differentiated only by their steroid use at a specified landmark time. Patients were followed up from the day of LT surgery (index date) until the earliest day of occurrence of PTDM, cessation due to death, or the end of the dataset as censored observations. Additional information and the study scheme are shown in Supplementary Methods 1.1, Supplementary Figure S1.

### Operational Definitions

The exposure of interest was steroid withdrawal, which is a dichotomous time-dependent variable. We defined steroids as the three corticosteroid medications commonly used after LT in South Korea; prednisolone/prednisone, methylprednisolone, and deflazacort [23].

In this study, PTDM was defined as a type 2 diabetes diagnosis along with a prescription for antidiabetic medication including insulin, with the date of the first prescription considered the occurrence of the outcome [24]. Steroid use was defined with a 30-day grace period, with maintenance as continuous use with a prescription refill within 30 days of the last fill date and withdrawal as no refill within 30 days. To prevent the inclusion of steroid use for other conditions, we considered only prescriptions where the diagnosis of “liver transplant status” (ICD-10-CM code of Z94.4) was confirmed on the same date as the prescription. Deceased individuals were identified by the absence of medical claims for over a year from their last visit. Lastly, we investigated whether steroid withdrawal compromised the goal of preventing allograft rejection, focusing on incidents occurring within 2 months of withdrawal [25]. Rejection events were defined as prescriptions of 500 mg or more of intravenous methylprednisolone, based on medical advice. A detailed explanation is provided in Supplementary Methods 1.2.

### Statistical Analysis

Data are summarized as frequencies with percentages or means with standard deviations. The chi-square test or Fisher’s exact test was used for categorical variables, while continuous variables were analyzed using Student’s t-test. Kaplan-Meier analysis was used to estimate the probability of outcomes at daily intervals with censoring. If the day of the outcome of interest in a patient’s record was within the analysis timeframe, the patient was censored on that day. For these matched cohorts, we measured the incidence of PTDM. The incidence rate was calculated by dividing the number of events by the total number of person years of follow-up, then multiplying by 1,000. We performed Cox proportional hazards regression analysis to describe the relative hazard of the outcomes, comparing the time-to-event rates with HRs with 95% Confidence Interval (CI). Statistical significance was set as less than 0.05. The proportional hazards assumption was verified using a log minus log plot. All analyses were conducted using the SAS software (version 9.4.2 (SAS Institute, Inc., Cary, NC, USA)).

## Results

### Patient Characteristics at Transplantation

**FIGURE 1:**
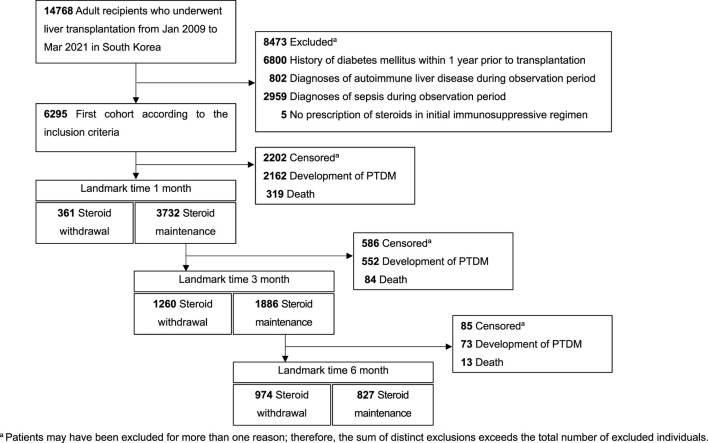
Flow diagram of cohort construction.

A total of 14,768 adult patients who underwent LT in South Korea between January 2009 and March 2021 were identified. For the analysis of incident PTDM, the study cohort included 6,295 patients with no history of diabetes (Figure 1). The mean age at LT was 53.0 years, with approximately 400–600 recipients annually. The majority were male (70.7%), and most underwent living donor LT (76.4%) (Table 1). The tacrolimus-based regimen for initial post-transplantation immunosuppression was dominant (94.9%), with the use of a cyclosporin-based regimen becoming infrequent in 2016.

**TABLE 1:** Baseline characteristics of study participants with liver transplantation.

Characteristics	No. (%)	*P* value
Total no. of patients	6,295 (100)	​
Age at LT, mean (SD), y	53.0 (15.2)	-
Male	4,451 (70.7)	<0.0001
Insurance type	​	<0.0001
National health insurance	5,945 (94.4)	​
Medical aid	350 (5.6)	​
Types of transplantation	​	<0.0001
Living donor LT	4,812 (76.4)	​
Deceased donor LT	1,483 (23.6)	​
Immunosuppression	​	<0.0001
Tacrolimus-based regimen	5,975 (94.9)	​
Cyclosporin A-based regimen	115 (1.8)	​
Other regimen	205 (3.3)	​
CCI score, mean (SD)	4.3 (2.2)	-
Comorbidities	​	<0.0001
Hypertension	841 (13.4)	​
Dyslipidemia	102 (1.6)	​
Osteoporosis	152 (2.4)	​
Congestive heart failure	304 (4.8)	​
Peripheral vascular disease	241 (3.8)	​
Chronic pulmonary disease	1,379 (21.9)	​
Rheumatologic disease	122 (1.9)	​
Renal disease	157 (2.5)	​

Abbreviations: CCI, charlson comorbidity index; LT, liver transplantation; n, number; SD, standard deviation; Y, year.

Among the patients followed up with, 41.5% withdrew from steroid use within 6 months after LT, and 44.5% were diagnosed with PTDM within the same period. The mean follow-up duration was 5.3 years (median 4.8 years; interquartile range 2.0–8.3 years). After the patients were classified based on steroid use and matched by propensity scores, the two groups were balanced at each landmark time. The patient characteristics before and after matching are presented in Supplementary Table S2.

### PTDM in LT Recipients at 1-Month Landmark Time

**FIGURE 2:**
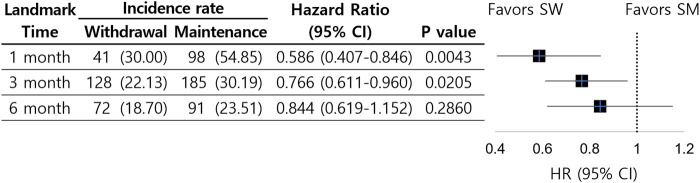
Incidence rate (per 1000 person-years) and adjusted hazard ratio for post-transplant diabetes mellitus according to steroid withdrawal timing at each landmark time after liver transplantation. Abbreviations: CI, confidence intervals; HR, hazard ratio; SM, steroid maintenance; SW, steroid withdrawal.

Among the initial cohort of 6,295 patients, 34.3% were diagnosed with PTDM within 1-month post-LT and 5.1% died. These patients were excluded from analysis at the 1-month landmark. The steroid maintenance group (hereafter SMG) included 3,732 patients, while the steroid withdrawal group (hereafter SWG) included 361 patients. After propensity score matching, each cohort group consisted of 351 patients. The incidence rate was 54.9 cases per 1,000 person-years in the SMG and 30.0 cases per 1,000 person-years in the SWG, indicating that the rate of new PTDM cases in the SMG was 1.8 times higher than that in the SWG (*P* < 0.00001). The Kaplan-Meier cumulative survival curves showed a higher risk of PTDM at every time point in the SMG than in the SWG. The log-rank test demonstrated that the observed difference in risk between the two groups was statistically significant (*P* = 0.0018). Cox proportional hazards model analysis revealed that at the 1-month landmark time, the SWG had a significantly lower risk of PTDM compared to the matched SMG (5.8% vs. 14.0%; HR, 0.59; 95% CI, 0.41–0.85) (Figure 2).

### PTDM in LT Recipients at 3-Month Landmark Time

Among the 3,732 uncensored patients at the 1-month landmark, 14.8% were diagnosed with PTDM within 3-month post-LT and 2.3% died. These patients were excluded from the 3-month landmark analysis, which included 1,886 patients in the SMG and 1,260 in the SWG. After propensity score matching, each cohort group consisted of 1,257 patients. The incidence rate was 30.2 cases per 1,000 person-years in the SMG and 22.1 cases per 1,000 person-years in the SWG, indicating that the rate of new PTDM cases in the SMG was 1.4 times higher than that in the SWG (*P* = 0.0006). The Kaplan-Meier cumulative survival curves showed a slightly higher risk of PTDM at every time point in the SMG than in the SWG. The log-rank test demonstrated that the observed difference in risk between the two groups was also statistically significant (*P* = 0.0073). Cox proportional hazards model analysis revealed that at the 3-month landmark time, the SWG had a significantly lower risk of PTDM compared to the matched SMG (5.1% vs. 7.4%; HR, 0.77; 95% CI, 0.61–0.96) (Figure 2).

### PTDM in LT Recipients at 6-Month Landmark Time

Among the 1,886 uncensored patients at the 3-month landmark, 3.9% were diagnosed with PTDM within 6-month post-LT and 0.7% died. These patients were excluded from the 6-month landmark analysis, which included 827 patients in the SMG and 974 in the SWG. After propensity score matching, each cohort comprised of 782 patients. The incidence rate were 23.5 cases per 1,000 person-years in the SMG and 18.7 cases per 1,000 person-years in the SWG; however, the difference was not statistically significant (*P* = 0.1159). The Kaplan-Meier cumulative survival curves did not show a clear difference in the risk of PTDM occurrence between the two groups at any time point. The log-rank test confirmed that the observed risk difference between the groups was not statistically significant (*P* = 0.1393). Cox proportional hazards model analysis showed that at the 6-month landmark time, the SWG had a lower risk of PTDM compared to the matched SMG, but this difference was not statistically significant (4.6% vs. 5.8%; HR, 0.84; 95% CI, 0.62–1.15) (Figure 2).

### Allograft Risk After Steroid Withdrawal

Although rejection is becoming less frequent in LT recipients, we monitored for allograft rejection to ensure the safety of steroid withdrawal. We examined the requirement for high-dose steroid pulse (≥500 mg/dose of prednisolone) as a treatment for acute cellular or humoral rejection within 2 months post-withdrawal. Two patients received such therapy, both of whom belonged to the 3-month landmark withdrawal group.

## Discussion

Minimizing exposure to immunosuppressive agents, especially steroids, in solid organ transplant recipients has been discussed for decades. However, no international consensus has been reached on its optimal timing or the appropriate withdrawal protocol. This was attributable to substantial heterogeneity in regimens and withdrawal schedules across LT studies. A systematic review of 16 randomized controlled trials (RCT) reported persistent ambiguity regarding the benefits and harms of steroid avoidance or withdrawal after LT, finding no significant differences in mortality, graft loss, or infection between steroid-free and steroid-containing regimens [26]. Regarding diabetes outcomes, a subgroup analysis yielded inconsistent results depending on the analytical approach: the fixed-effects model showed a significant reduction in risk (Relative Risk (RR), 0.81; 95% CI, 0.66–0.99), whereas the random-effects model did not (RR, 0.82; 95% CI, 0.64–1.07) [26]. Furthermore, substantial risk of bias and wide variability in withdrawal timing (64–180 days) among the included trials limited the reliability of comparisons across specific time points.

In a recent RCT comparing early withdrawal (2 weeks ±3 days) with later withdrawal (3 months ±2 weeks), the incidence of new-onset diabetes was significantly higher in the earlier-withdrawal group (23.3% vs. 5.5%; *P* = 0.008), likely attributable to intensified tacrolimus exposure [27]. However, that study was limited by a relatively short follow-up period of 1 year, restricting evaluation of long-term outcomes.

Despite these uncertainties, efforts to reduce steroid use have continued, supported by the liver’s unique immune privilege, which may allow for safer minimization of immunosuppression [28]. This paradigm reflects a shift in focus from short-term graft survival toward long-term patient-centered outcomes.

In this context, our study provided robust evidence using a large cohort with up to 13 years of follow-up. We demonstrated that early steroid withdrawal reduced the risk of PTDM by 23%–41%. In the era of predominantly tacrolimus-based regimens, withdrawal within 3 months appeared to be a safe and effective strategy. Notably, continuing steroids more than 3 months offered no additional benefit even when withdrawal occurred before 6 months, underscoring the importance of discontinuing steroids as early as feasible within the first 3 months after transplantation. By applying a landmark design, we minimized immortal time bias and achieved an accurate assessment of time-dependent drug effects. In addition, the strict delineation of protocol-driven steroid exposure from therapeutic administration for comorbidities allowed us to mitigate confounding by indication. Combined with the external validity of a nationwide dataset reflecting real-world clinical practice, this study provides actionable evidence to guide immunosuppressive management and prognosis after LT.

Several limitations should be acknowledged. As steroid withdrawal may have reflected centre-led clinical decision-making, residual confounding from unmeasured factors cannot be excluded, despite landmark-specific propensity score matching to balance measured covariates [29]. Reliance on claims data also introduced the possibility of coding errors, and clinical parameters such as glycemic profiles and body mass index were unavailable. Our design also limited our ability to examine pre-transplant risk factors for developing PTDM or to evaluate the clinical rationale underlying decisions regarding steroid withdrawal, which may vary across institutions or among individual clinicians. This study addressed only the duration of steroid use without considering cumulative dosage or relative potency. Steroid withdrawal may have been accompanied by intensification or substitution with other immunosuppressants (e.g., calcineurin inhibitors, antimetabolites). Nevertheless, we prioritized steroid withdrawal as the primary exposure of interest, consistent with the conclusion of Van Hooff et al (2004) that avoidance or early withdrawal represents the best preventive strategy against diabetes [30]. Finally, as our cohort was derived exclusively from the South Korean population, caution is warranted when extrapolating these results to other geographic regions with different baseline patient characteristics or genetic backgrounds.

A substantial proportion of recipients developed hyperglycemia or diabetes within the first month after LT. These early cases were excluded from the landmark analyses for methodological reasons. Yet, accumulating evidence suggests that this early period represents a clinically important metabolic phase. Studies in kidney and other solid-organ transplant populations indicated that early post-transplant hyperglycemia was associated with adverse outcomes, including infections, rehospitalizations, and graft dysfunction [31, 32]. Together, these findings underscore the need for dedicated investigations focused on this early post-transplant period.

In conclusion, this nationwide cohort study showed that early steroid withdrawal after LT was associated with a lower risk of PTDM, with the greatest benefit observed when steroids were discontinued within 3 months. Using a landmark-based approach in a large, real-world population, our findings provide time-specific evidence relevant to contemporary tacrolimus-based practice. These results support earlier consideration of steroid minimization and may help inform clinical decision-making aimed at improving long-term metabolic outcomes in LT recipients.

## Data Availability

The data analyzed in this study is subject to the following licenses/restrictions: Data were obtained from the Health Insurance Review and Assessment Service (HIRA) under license and are not publicly available. Access requires administrative approval, and all patient information was anonymized in accordance with the Personal Information Protection Act. Requests to access these datasets should be directed to Health Insurance Review and Assessment Service (HIRA).
