# Supplementary material for: The Clinical Impact of Early Steroid Withdrawal on Diabetes Mellitus After Liver Transplantation: A Population-Based Cohort Study
Source: Transpl Int. 2026 Feb 12;39:15432. doi: 10.3389/ti.2026.15432 (PMC12935688; doi:10.3389/ti.2026.15432)
Supplement: Supplementary file 1 [file DataSheet1.pdf]

Supplementary Material

Capsule Sentence Summary

Graphical Abstract

Supplemental Methods

1.1 Landmark analysis based on steroid use

1.2 Operational definitions

Figure S1. The flow diagram of study scheme

Table S1. ICD code definitions

Table S2. Patient characteristics before and after propensity score matching 6 months after liver transplantation

This supplementary material has been provided by the authors to give readers additional information about their work.

Capsule Sentence Summary

Early corticosteroid withdrawal after liver transplantation significantly reduced post-transplant diabetes risk without increasing rejection, with discontinuation within 3 months emerging as the most effective and safe window in tacrolimus-based immunosuppression. It supports timely steroid tapering to improve long-term metabolic outcomes.

Graphical Abstract

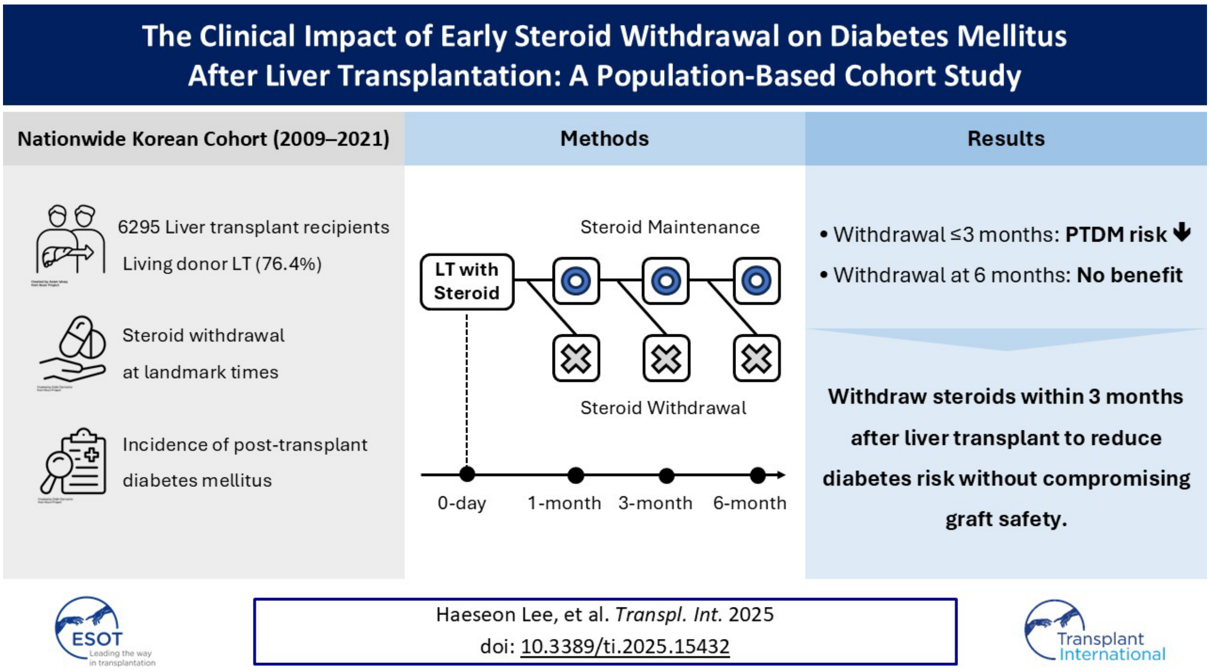

Supplemental Methods

1.1 Landmark analysis based on steroid use

Landmark analysis is a type of survival analysis that classifies patients based on interim responses that occur during follow-up only for subjects alive at the landmark time[1]. We set 1-, 3-, and 6-months post-transplantation as landmark times. These specific times were decided based on medical advice from a clinician specializing in LT in South Korea, as they are clinically meaningful time points for making medication adjustments in the management of LT recipients. At each time point, the patients were categorized into 'steroid withdrawal' or 'steroid maintenance' groups according to their steroid use status from the previous landmark time to the current landmark time.

To address potential differences between the groups that could lead to confounding by indication, the propensity score (PS), which represents the conditional probability of continuing steroids, was introduced[2]. We calculated PS using logistic regression models at each landmark time point based on the latest covariates, including comorbidities (hypertension, dyslipidemia, osteoporosis, congestive heart failure, peripheral vascular disease, renal disease, chronic pulmonary disease, and rheumatic disease) [3] and HCV infections[4], alongside demographics (age, sex, LT year, and type of liver donor) and immunosuppressive regimen. Subsequently, we performed 1:1 matching of patients from the steroid withdrawal group with those from the steroid maintenance group, utilizing their individual PS. This matching generated two cohorts of patients whose only remaining difference, in theory, was their steroid use at a given landmark time.

The exposure of interest was steroid withdrawal, which is a dichotomous time-dependent variable. We defined steroids as the three corticosteroid medications commonly used after LT in South Korea; prednisolone/prednisone, methylprednisolone, and deflazacort. Steroid use is described in the operational definition section. Patients were followed up from the day of LT surgery (index date) until the earliest day of occurrence of PTDM, cessation due to death, or the end of the dataset as censored observations.

Figure S1. The flow diagram of study scheme

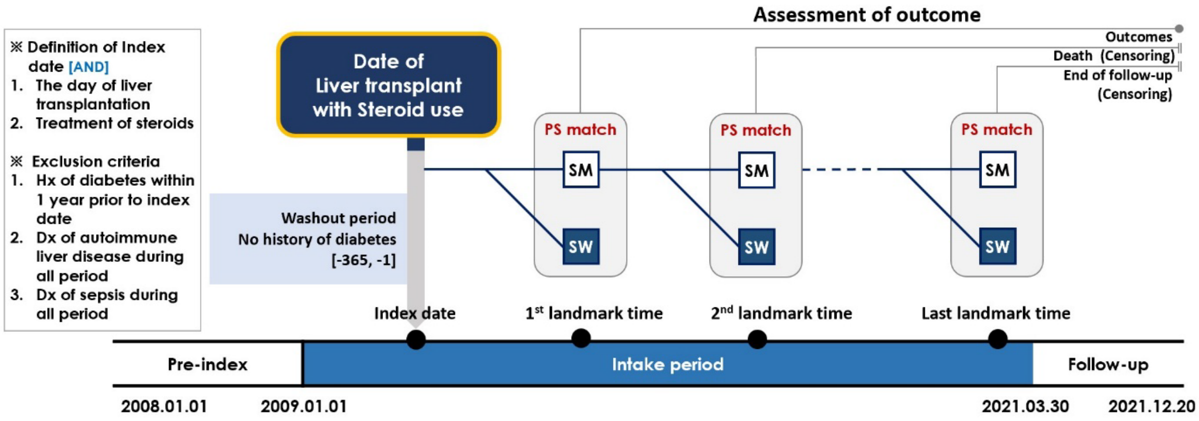

Dx, diagnosis; Hx, history; PS, propensity score; SM, steroid maintenance; SW, steroid withdrawal.

1.2 Operational definitions

The exposure of interest was steroid withdrawal, which is a dichotomous time-dependent variable. We defined steroids as the three corticosteroid medications commonly used after LT in South Korea; prednisolone/prednisone, methylprednisolone, and deflazacort [5].

The dependent variable and outcome of this study was PTDM in LT recipients. PTDM is a form of type 2 diabetes mellitus characterized by relative insulin deficiency due to impaired insulin production or increased insulin resistance[6]. It was managed according to the treatment guidelines established by the American Diabetes Association (ADA) and the European Association for the Study of Diabetes (EASD)[7]. Although PTDM is usually diagnosed using blood glucose tests as specified by international diagnostic criteria[8], the HIRA dataset lacks this information. Therefore, we used diagnostic codes and prescriptions based on various retrospective studies[9]. and medical advice to identify patients with PTDM. In this study, PTDM was defined as the presence of a type 2 diabetes diagnosis (ICD-10-CM code E11-E14) along with a prescription for antidiabetic medication, with the date of the first prescription considered as the occurrence of the outcome. The list of antidiabetics includes all medications under the A10 category of the anatomical therapeutic chemical (ATC) code developed and maintained by the World Health Organization (WHO). This category covers both oral antidiabetic agents and insulin injections, as insulin therapy is frequently used to manage hyperglycemia in the early post-transplant period[10].

Steroid use was defined as a 30-day grace period. Steroid maintenance was defined as the continuous use of medication with a prescription refill within 30 days of the last fill date, starting from the index date. The last filling date was calculated as the prescription filling date and the number of days supplied[11]. Conversely, steroid withdrawal was defined as the absence of a prescription refill within 30 days of the last fill date, marking that date as the end of medication use. To prevent the inclusion of steroid use for other conditions, we considered only prescriptions where the diagnosis of “liver transplant status” (ICD-10-CM code of Z94.4) was confirmed on the same date as the prescription.

In the landmark analysis, we needed to identify deceased individuals to exclude them because the analysis only included patients who survived until the landmark period [1]. As the HIRA dataset reported only in-hospital deaths, we considered patients without any medical claims

for over a year from their last visit as deceased, which was confirmed by a clinician, and defined the date of death as the discharge date from the last observed claim[12]. This one-year period was reasonable for LT recipients who required lifelong management and regular outpatient visits, even if the graft was well engrafted. Additionally, studies on renal transplantation have shown that using this definition of death results in over 95% agreement with national death registries[13].

We examined whether steroid withdrawal compromised the goal of steroid use in preventing allograft rejection. To ensure that rejection events directly associated with steroid withdrawal were captured, we focused on incidents occurring within 2 months of the withdrawal date. Based on medical advice that high-dose steroid use is a more reliable indicator of rejection than diagnostic codes in clinical settings, we defined rejection events as prescriptions of 500 mg or more of intravenous methylprednisolone.

Table S1. ICD code definitions

Certain variables are defined based on the presence or absence of International Classification of Diseases (ICD) codes in patient claims data. The ICD code information for these variables is provided below. Unless stated otherwise, ICD-10 codes were utilized.

Variables	ICD codes
Diabetes mellitus	E10-E14<br>E11-E14 was used for type 2 diabetes
Autoimmune liver disease	K75.4, K74.3-K74.5, K83.0
Sepsis	A40-A41, R65
HCV infection	B17.1, B18.2
Hypertension	I10, I15
Dyslipidemia	E78
Osteoporosis	M80-M81
Congestive heart failure	I09.9, I11.0, I13.0, I13.2, I25.5, I42.0, I42.5-I42.9, I43, I50, P29.0
Peripheral vascular disease	I70-I71, I73.1, I73.8-I73.9, I77.1, I79.0, I79.2, K55.1, K55.8, Z95.8-Z95.9
Chronic pulmonary disease	J40-J47, J60-J67, J684, J701, J703, I27.8-I27.9
Rheumatic disease	M05-M06, M31.5, M32-M34, M35.1, M35.3, M36.0
Renal disease	N18-N19, I12.0, I13.1, N03.2-N03.7, N05.2-N05.7, N25.0, Z49.0-Z49.2, Z94.0, Z99.2
liver transplant status	Z94.4

Table S2. Patient characteristics before and after propensity score matching 6 months after liver transplantation; 2,787 were diagnosed with PTDM and 416 deaths occurred by 6 months after liver transplantation

Characteristics	Crude			Matched		
	SWG	SMG	SMD(%)†	SWG	SMG	SMD(%)†
**Number of recipients**, n	**974**	**827**		**782**	**782**	
**Age at LT**, y(mean±SD)	52.0±10.2	52.6±9.9	-6.0	52.3±10.0	52.5±10.0	-1.7
**Male**, n(%)	672 (69.0)	591 (71.5)	-5.4	556 (71.1)	559 (71.5)	-0.9
**Types of transplantation**, n(%)			7.3			0.9
Living donor LT	711 (73.0)	630 (76.18)		589 (75.3)	592 (75.7)	
Deceased donor LT	263 (27.0)	197 (23.82)		193 (24. 7)	190 (24.3)	
**Immunosuppression**, n(%)			-2.5			2.1
Tacrolimus-based regimen	940 (96.5)	799 (96.6)		756 (96.7)	755 (96.6)	
Cyclosporin-based regimen	10 (1.0)	10 (1.2)		10 (1.3)	9 (1.2)	
Others	24 (2.5)	18 (2.2)		16 (2.1)	18 (2.3)	
**CCI score,** (mean±SD)	4.2±2.1	4.2±2.2		4.2±2.2	4.2±2.1	
**Comorbidities‡,** n(%)						
Hypertension	474 (48.7)	359 (43.4)	10.6	348 (44.5)	347 (44.4)	0.3
Dyslipidemia	90 (9.2)	90 (10.9)	-5.5	76 (9.7)	81 (10.4)	-2.1
Osteoporosis	120 (12.3)	96 (11.6)	2.2	88 (11.3)	90 (11.5)	-0.8
HCV infection	44 (4.5)	44 (5.3)	-3.7	37 (4.7)	40 (5.1)	-1.8

**Characteristics**	**Crude**			**Matched**		
	**SWG**	**SMG**	**SMD(%)†**	**SWG**	**SMG**	**SMD(%)†**
**Number of recipients**, n	**974**	**827**		**782**	**782**	
Congestive heart failure	66 (6.8)	44 (5.3)	6.1	44 (5.6)	42 (5.4)	1.1
Peripheral vascular disease	83 (8.5)	89 (10.8)	-7.6	76 (9.7)	66 (8.4)	4.5
Chronic pulmonary disease	319 (32.8)	200 (24.2)	19.1	207 (26.5)	199 (25.5)	2.3
Rheumatologic disease	19 (2.0)	17 (2.1)	-0.8	14 (1.8)	15 (1.9)	-1.0
Renal disease	68 (7.0)	75 (9.1)	-7.7	62 (7.9)	59 (7.5)	1.4

CCI, charlson comorbidity index; HCV, hepatitis C virus; LT, liver transplantation; SD, standard deviation; SMD, standardized mean difference; SMG, steroid maintenance group; SWG, steroid withdrawal group.

† SMD greater than 10% was a threshold indicating group imbalance.

‡ History of comorbidities was identified by diagnosis within 1-year prior to each landmark time.

Reference

1. Dafni U. Landmark analysis at the 25-year landmark point. *Circulation: Cardiovascular Quality and Outcomes*. 2011;4(3):363-371.
2. Austin PC. An Introduction to Propensity Score Methods for Reducing the Effects of Confounding in Observational Studies. *Multivariate Behav Res*. May 2011;46(3):399-424. doi:10.1080/00273171.2011.568786
3. Li D-W, Lu T-F, Hua X-W, et al. Risk factors for new onset diabetes mellitus after liver transplantation: A meta-analysis. *World Journal of Gastroenterology: WJG*. 2015;21(20):6329.
4. Chowdhury TA, Wahba M, Mallik R, et al. Association of British Clinical Diabetologists and Renal Association guidelines on the detection and management of diabetes post solid organ transplantation. *Diabetic Medicine*. 2021;38(6):e14523.
5. Park J, Joo D, Kim J. Liver transplantation and new life. The Korean Society for Transplantation. Accessed June 30, 2022. https://mykst.org/wp-content/uploads/2024/06/%EA%B0%84%EC%9D%B4%EC%8B%9D%EA%B3%BC-%EC%83%88%EB%A1%9C%EC%9A%B4-%EC%82%B6-%EB%82%B4%EC%A7%80.pdf
6. Zaccardi F, Webb DR, Yates T, Davies MJ. Pathophysiology of type 1 and type 2 diabetes mellitus: a 90-year perspective. *Postgrad Med J*. Feb 2016;92(1084):63-9. doi:10.1136/postgradmedj-2015-133281
7. Yun Y, Kang ES. Management of Diabetes in Organ Transplant Patients. *Journal of Korean Diabetes*. 2014;15(3)
8. Pham P-T, Sarkar M, Pham P-M, Pham P-C. Diabetes mellitus after solid organ transplantation. 2016;
9. Lawendy B, Srinathan S, Kotha S, et al. Systematic review and meta-analysis of post-transplant diabetes mellitus in liver transplant recipients. *Clin Transplant*. Jul 2021;35(7):e14340. doi:10.1111/ctr.14340
10. Hecking M, Haidinger M, Döller D, et al. Early basal insulin therapy decreases new-onset diabetes after renal transplantation. *Journal of the American Society of Nephrology*. 2012;23(4):739-749.
11. Nielsen LH, Løkkegaard E, Andreasen AH, Keiding N. Using prescription registries to define continuous drug use: how to fill gaps between prescriptions. *Pharmacoepidemiol*

Drug Saf. Apr 2008;17(4):384-8. doi:10.1002/pds.1549

12. Gil E, Kim JM, Jeon K, et al. Recipient age and mortality after liver transplantation: a population-based cohort study. *Transplantation*. 2018;102(12):2025-2032.

13. Park S, Kim M, Kim JE, et al. Characteristics of kidney transplantation recipients over time in South Korea. *The Korean Journal of Internal Medicine*. 2020;35(6):1457.
